# Does shortage of GPs matter? A cross-sectional study of practice population life expectancy

**DOI:** 10.3399/BJGP.2023.0195

**Published:** 2024-04-16

**Authors:** Richard Baker, Louis S Levene, Christopher Newby, George K Freeman

**Affiliations:** Department of Population Health Sciences, University of Leicester, Leicester.; Department of Population Health Sciences, University of Leicester, Leicester.; School of Medicine, University of Nottingham, Nottingham.; Department of Primary Care and Public Health, School of Public Health, Faculty of Medicine, Imperial College London, London.

**Keywords:** continuity of patient care, cross-sectional studies, health inequities, health workforce, life expectancy, primary health care

## Abstract

**Background:**

There are not enough GPs in England. Access to general practice and continuity of care are declining.

**Aim:**

To investigate whether practice characteristics are associated with life expectancy of practice populations.

**Design and setting:**

A cross-sectional ecological study of patient life expectancy from 2015–2019.

**Method:**

Selection of independent variables was based on conceptual frameworks describing general practice’s influence on outcomes. Sixteen non-correlated variables were entered into multivariable weighted regression models: population characteristics (Index of Multiple Deprivation, region, % White ethnicity, and % on diabetes register); practice organisation (total NHS payments to practices expressed as payment per registered patient, full-time equivalent fully qualified GPs, GP registrars, advanced nurse practitioners, other nurses, and receptionists per 1000 patients); access (% seen on the same day); clinical performance (% aged ≥45 years with blood pressure checked, % with chronic obstructive pulmonary disease vaccinated against flu, % with diabetes in glycaemic control, and % with coronary heart disease on antiplatelet therapy); and the therapeutic relationship (% continuity).

**Results:**

Deprivation was strongly negatively associated with life expectancy. Regions outside London and White ethnicity were associated with lower life expectancy. Higher payment per patient, full-time equivalent fully qualified GPs per 1000 patients, continuity, % with chronic obstructive pulmonary disease having the flu vaccination, and % with diabetes with glycaemic control were associated with higher life expectancy; the % being seen on the same day was associated with higher life expectancy in males only. The variable aged ≥45 years with blood pressure checked was a negative predictor in females.

**Conclusion:**

The number of GPs, continuity of care, and access in England are declining, and it is worrying that these features of general practice were positively associated with life expectancy.

## Introduction

Life expectancy at birth in England increased throughout the 20th century, but improvement has now stalled.^1^^,^^2^ Although similar slowdowns have occurred in comparable high-income countries, the UK has fallen down the global rankings.^2^^,^^3^ These slowdowns pre-date the COVID-19 pandemic, and the explanation is not fully established. A review of mortality trends in the UK highlighted wide gaps between the more and less affluent, a greater slowdown in rates among females, and an increase in mortality among those aged 45–49 years.^4^ A combination of factors is probably responsible, including austerity, increasing socioeconomic inequalities, the social determinants of health, the slowing of mortality improvements in cardiovascular disease management, and a waning of the healthy immigrant effect.^5^^–^^7^

There is only limited evidence that general practices, or their funding or staffing, affect population mortality in England,^8^ and none, to the authors’ knowledge, about their influence on the slowdown of life expectancy improvements. Investigation of variations in life expectancy between practices might suggest how primary care should respond. Between the quinquennia 2013–2017 and 2015–2019, female life expectancy declined in 43.0% of practices and male life expectancy declined in 39.7%.^9^ The current study set out to investigate whether practice characteristics are associated with life expectancy.^10^ The hypotheses were that increased life expectancy is associated with:

- increased numbers of practice staff (hypothesis 1);
- improved access and continuity (hypothesis 2); and
- better clinical care in practices (hypothesis 3).

**Table:** How this fits in

Primary care in England is under severe pressure because of a shortage of GPs and growing morbidity in the population, leading to declines in access and continuity of care. A cross-sectional study was undertaken to investigate associations between population and practice characteristics and the average life expectancy of practice populations. Higher payments per patient, more full-time equivalent fully qualified GPs per 1000 patients, higher continuity, and better performance on two of four measures of clinical care were associated with higher life expectancy in both males and females, and better on-the-day access was associated with higher life expectancy in males. The current study could not establish causation, but the findings are consistent with international evidence about the mechanisms of primary care and should trigger concern about the effect of the current general practice crisis on population health in England.

## Method

### Overview

The study was cross-sectional, ecological, and included practices in England with life expectancy data in the National General Practice Profiles system^9^ when the data were extracted (August 2022). Practices included in the National General Practice Profiles system had to be recognised by the NHS’s data service^11^ and have a list size of ≥750 patients. All data were summary statistics about practices and their populations (Supplementary Box S1).

### Dependent variables

The two dependent variables were period life expectancy for patients in the quinquennium 2015–2019. Period life expectancy indicates the average number of years someone born in the period of interest can be expected to live and is calculated from age-specific mortality rates using data collected by the Office for National Statistics.^9^ As general practices have relatively small populations, annual measures of mortality can mislead; therefore, values are calculated over 5-year periods (see Supplementary Box S2 for further details).

### Independent variables

Selection of potential independent variables was driven by two conceptual frameworks: the SEARCH framework, in which population factors are important determinants of health outcomes,^12^ and a framework describing the mechanisms of primary care that influence population mortality, developed using evidence from low-, middle-, and high-income countries.^13^ A landmark review in 2005 described six mechanisms of primary care accounting for its beneficial effects on population health.^10^ The authors of the current study’s new framework categorises 23 mechanisms into five groups:

- organisation;
- access;
- comprehensiveness;
- clinical care; and
- the therapeutic relationship.

Comprehensiveness, the lifelong care of all individuals, is dependent on health system policies and was excluded as the study concerns a single health system. Potential variables with correlations of ≥0.4 with other variables were excluded (Supplementary Box S3 and Supplementary Tables S1–S3).

The measure of deprivation used in the current study was the 2019 version of the Index of Multiple Deprivation (IMD),^14^ which used 39 indicators from seven domains (income, employment, health, education, housing, crime, and environment). Practice IMD values were obtained from the National General Practice Profiles system;^9^ these were estimated by taking a weighted average of the IMD scores for each lower-level super output area in which a given practice had registered patients. Practices’ NHS commissioning region^15^ was included as there could be regional variations of health services’ provision and life expectancy.

Data from the Quality and Outcomes Framework (QOF),^16^ collected from general practice records, provided practice list sizes, disease prevalence, and clinical performance variables (Supplementary Box S4). Disease prevalences were used to represent population morbidity. Mean prevalence was calculated from the annual rates for each year 2015 to 2019, with conditions excluded if their definitions changed during this period. In the current study, the authors initially selected conditions that could plausibly affect whole-population life expectancy. For example, chronic obstructive pulmonary disease (COPD) was included rather than asthma because there are many more deaths from COPD than from asthma.^17^ The selected conditions were coronary heart disease (CHD), stroke, diabetes, hypertension, COPD, and cancer. The prevalences of all these conditions were combined to create another potential measure of morbidity and the authors also considered the percentage of patients reporting having a long-standing condition, derived from the General Practice Patient Survey (GPPS) (https://www.gp-patient.co.uk). However, only diabetes prevalence was retained, as all the other morbidity variables were highly correlated with each other or with ethnicity variables (Supplementary Table S2).

Variables relating to clinical care performance that could plausibly affect life expectancy were derived from QOF indicators that were defined consistently throughout 2015 to 2019. After eliminating highly correlated variables, four were retained — the percentages of patients:

- aged ≥45 years who had blood pressure recorded in the preceding 5 years;
- with COPD who were vaccinated against flu;
- with diabetes whose last International Federation of Clinical Chemistry HbA1c was ≤59 mmol/mol; and
- with CHD on antiplatelet or anticoagulant therapy.

Data from the GPPS between 2015 and 2019 provided measures of ethnicity and patient reports of access and continuity. GPPS questionnaires are sent annually to samples of patients in every practice nationwide (Supplementary Box S5). Samples are weighted to resemble population characteristics within each practice, accounting for factors that include age, gender, ethnicity, and marital status. The survey was substantially revised in 2018, with changes to some variable definitions and to the sample surveyed. Data, therefore, from the first 3 years only (2015–2017) were used in the current study. The ethnicity categories used were the five bandings nationally recommended (White, Asian, Black, mixed, and other).^18^ The GPPS did not report the exact figure if the percentage of people in a particular practice belonging to an ethnic group was <0.5%. These values were coded as 0%. As ethnicity categories were strongly correlated with each other, only White ethnicity was retained.

After checking for correlation between variables, for the access variable in the current study the percentage of patients booking an appointment who were seen on the same day was selected (Supplementary Table S1). For the continuity variable, the product of the percentage of people who had a doctor they preferred to see and the combined percentage of those reporting being able to see that doctor always, almost always, or a lot of the time was used.^19^ Only 2015 data were used for continuity because of the cumulative problem of missing data for this variable over several years. GPPS-reported rates of smoking were excluded in the current study as these were correlated with IMD 2019.

Funding was expressed as NHS payments per patient (Supplementary Box S6).^20^ General practice workforce data are published using a standardised reporting system.^21^ Information is provided on four staff groups: GPs, nurses, administrative staff, and direct patient care staff (such as dispensers and assistants), published as both head counts and full-time equivalents (FTEs); full-time work was defined as 37.5 h per week.^21^ FTEs were used in the current study. Direct patient care staff were excluded from the study as these data were incomplete. From within the other groups, fully qualified GPs, GP registrars, advanced nurse practitioners (ANPs), other practice nurses (excluding ANPs), and receptionists were selected. GP registrars and ANPs consult with patients and may influence life expectancy. Receptionists were included as fewer could affect access to clinical staff.

### Analyses

Descriptive statistics were calculated before performing multivariable analyses, in which weighted regression models were fitted with practice list size as the weights to allow for the differential effect of differently sized practices. Cases with missing values were omitted.

Model performance was assessed for collinearity, homogeneity of variance, normality of residuals, posterior predictive check, and Akaike and Bayesian information criterion. All analyses were performed in R Studio 2022.02.3 (https://www.r-project.org).

### Sensitivity analyses

The authors undertook univariable regressions of the independent variables for use in any future systematic reviews (Supplementary Table S4). To check for the effect of outliers, both unweighted ordinary least squares regression and robust regression models were fitted. Both these included list sizes, which no longer provided the weights. Weighted models were also fitted in which each of the other ethnicity groups replaced White ethnicity, and, for White ethnicity, in which unreported values were replaced with 0.5% instead of 0%.

## Results

### Descriptive statistics

The National General Practice Profiles resource included 6553 practices, of which 6489 (99.0%) had life expectancy data published for either males or females and formed the study population. Of these 6489 practices, 6477 had data for females and 6439 for males. Complete data for all variables included in the study analyses were available for females in 5875 practices (89.7% of all practices) and for males in 5742 (87.6% of all practices). The continuous variables’ descriptive statistics are in Table 1 and the numbers of practices in each NHS region in Table 2.

**Table 1.** Descriptive statistics of the continuous variables included in the final models (*n* = 6489 practices in England, 2015–2019)

**Variable**	***n***	**Missing, *n***	**Mean (SD)**	**Median (IQR)**
**Dependent variables**				
Female life expectancy, years	6477	12	83.3 (2.1)	83.4 (81.9–84.8)
Male life expectancy, years	6430	59	79.4 (2.3)	79.6 (77.8–81.1)

**Independent variables**				
Population				
IMD 2019 of practice populations	6481	8	23.4 (11.6)	21.4 (13.8–31.2)
Per cent of the practice population on the diabetes register	6299	190	4.8 (1.5)	4.6 (3.9–5.4)
Per cent of the practice population who are White ethnicity	6442	47	83.8 (21.4)	93.8 (78.4–97.8)
Per cent of the practice population who are mixed ethnicity	6442	47	1.2 (1.33)	0.7 (0.2–1.6)
Per cent of the practice population who are Asian ethnicity	6442	47	8.6 (14.3)	2.8 (0.9–9.1)
Per cent of the practice population who are Black ethnicity	6442	47	3.2 (5.8)	0.8 (0.0–3.6)
Per cent of the practice population who are other ethnicity	6442	47	3.2 (4.6)	1.2 (0.4–4.2)
List size	6288	201	8334 (4809)	7476 (4797–10 840)
Organisation				
Annual funding/patient, £	6455	34	154.8 (49.4)	141.6
FTE fully qualified GPs/1000 patients	6327	162	0.5 (0.2)	0.5
FTE GP registrars/1000 patients	6327	162	0.1 (0.1)	0.00
FTE ANPs/1000 patients	6224	265	0.05 (0.08)	0.00
FTE nurses excluding ANPs/1000 patients	6194	295	0.21 (0.11)	0.2
FTE receptionists/1000 patients	6391	98	0.6 (0.3)	0.6 (0.4–0.7)
Access				
Per cent seen the same day	6438	51	36.9 (13.5)	34.8 (26.8–45.7)
Therapeutic relationship				
Per cent receiving continuity of care	6195	294	32.0 (0.13)	30.7 (22.3–40.2)
Clinical care				
BP002, per cent BP checked in last 5 years	6281	208	90.7 (2.9)	91.0 (89.5–92.5)
COPD007, per cent with COPD vaccinated against flu	6270	219	80.2 (5.1)	80.5 (77.1–83.6)
DM007, per cent with diabetes in whom the last IFCC-HbA1c was ≤59 mmol/mol	6288	201	60.9 (6.1)	61.1 (57.1 – 64.0)
CHD005, per cent with CHD on antiplatelet therapy or an anticoagulant	6287	202	92.0 (3.52)	92.4

*ANP = advanced nurse practitioner. BP = blood pressure. CHD = coronary heart disease. COPD = chronic obstructive pulmonary disease. FTE = full-time equivalent. IFCC = International Federation of Clinical Chemistry. IMD = Index of Multiple Deprivation. IQR = interquartile range. SD = standard deviation.*

**Table 2.** Numbers of practices in each NHS commissioning region (*n* = 6481)

**Region**	**Number of practices**	**Percentage of total**
**East of England**	657	10.1
**London**	1176	18.1
**Midlands**	1293	20.0
**North East and Yorkshire**	994	15.3
**North West**	976	15.1
**South West**	552	8.5
**South East**	833	12.9
**Unknown**	8	0.1
**Total**	6481	100.0

### Multivariable regressions

In the weighted multivariable regression (Table 3), population variables explained most of the variation. As expected, life expectancy was lower in practices with higher deprivation. The London region had the highest life expectancy.

Features of general practices also predicted life expectancy, but less powerfully. Higher payments per patient and more fully qualified GPs per 1000 patients were associated with higher life expectancy. However, numbers of GP registrars, ANPs, and receptionists did not predict life expectancy (hypothesis 1). Increased numbers of practice nurses excluding ANPs were associated with lower life expectancy (Table 3).

**Table 3.** Weighted regression models with female and male life expectancy as the dependent variables, list size being used as the weights^a^

**Independent variables**	**Life expectancy, females, 2015 to 2019**			**Life expectancy, males, 2015 to 2019**		

	**Estimate**	**95% CI**	***P*-value**	**Estimate**	**95% CI**	***P*-value**
**(Intercept)**	87.43	86.26 to 88.61	**<0.001**	81.31	80.17 to 82.45	**<0.001**

**Population characteristics**						
Deprivation (IMD 2019)	−0.12	−0.12 to −0.12	**<0.001**	−0.15	−0.16 to −0.15	**<0.001**
White ethnicity	−0.013	−0.02 to −0.01	**<0.001**	−0.007	−0.01 to −0.00	**<0.001**
Morbidity (% on diabetes register)	−0.036	−0.06 to −0.01	**0.006**	−0.011	−0.04 to 0.01	0.377
NHS regions compared with London region						
South West	−0.76	−0.89 to −0.63	**<0.001**	−0.98	−1.10 to −0.85	**<0.001**
South East	−0.85	−0.96 to −0.74	**<0.001**	−0.89	−1.00 to −0.78	**<0.001**
Midlands	−1.45	−1.56 to −1.35	**<0.001**	−1.50	−1.60 to −1.39	**<0.001**
East of England	−1.00	−1.11 to −0.88	**<0.001**	−0.92	−1.04 to −0.81	**<0.001**
North West	−1.78	−1.90 to −1.66	**<0.001**	−1.51	−1.63 to −1.39	**<0.001**
North East and Yorkshire	−1.59	−1.71 to −1.47	**<0.001**	−1.58	−1.70 to −1.46	**<0.001**

**Organisation**						
Mean unweighted funding/patient	0.0051	0.00 to 0.01	**<0.001**	0.0064	0.01 to 0.01	**<0.001**
Mean FTE fully qualified GPs/1000 patients	0.57	0.37 to 0.77	**<0.001**	0.50	0.31 to 0.70	**<0.001**
FTE GP registrars/1000 patients	0.16	−0.20 to 0.51	0.386	0.18	−0.16 to 0.52	0.290
FTE ANPs/1000 patients	−0.12	−0.54 to 0.30	0.585	0.17	−0.24 to 0.58	0.413
FTE nurses/1000 patients excluding ANPs	−0.64	−0.97 to −0.32	**<0.001**	−0.81	−1.12 to −0.49	**<0.001**
FTE receptionists/1000	−0.082	−0.21 to 0.05	0.228	0.03	−0.10 to 0.16	0.641

**Access**						
Seen on the same day	0.0019	−0.00 to 0.00	0.085	0.0023	0.00 to 0.00	**0.029**

**Therapeutic relationship**						
Continuity of care	0.0027	0.00 to 0.00	**0.016**	0.0028	0.00 to 0.00	**0.009**

**Clinical care**						
BP002 % patients ≥45 years of age who have BP recorded	−0.030	−0.04 to −0.02	**<0.001**	−0.011	−0.02 to −0.00	**0.036**
DM007 % patients with diabetes in whom the last IFCC-HbA1c was ≤59 mmol/mol	0.034	0.03 to 0.04	**<0.001**	0.030	0.02 to 0.04	**<0.001**
COPD007 % patients with COPD who had influenza immunisation	0.0086	0.00 to 0.02	**0.012**	0.016	0.01 to 0.02	**<0.001**
CHD005 % patients with CHD on antiplatelet therapy	−0.0004	−0.01 to 0.01	0.930	−0.0006	−0.01 to 0.01	0.905
Observations		5769			5726	
R^2^/R^2^ adjusted		0.718/0.717			0.789/0.788	

a P*-values in bold are statistically significant. ANPs = advanced nurse practitioners. BP = blood pressure. CHD = coronary heart disease. COPD = chronic obstructive pulmonary disease. FTE = full-time equivalent. IFCC = International Federation of Clinical Chemistry. IMD = Index of Multiple Deprivation.*

Higher percentages of appointments on the same day only significantly positively predicted male life expectancy, but higher continuity predicted higher life expectancy in both males and females (hypothesis 2) (Table 3).

Of the clinical care variables, increased flu vaccination in COPD and better control in diabetes were weak positive predictors of life expectancy, whereas increased recording of blood pressure in those aged ≥45 years was a negative predictor (hypothesis 3) (Table 3).

Residuals from all models were approximately normally distributed. Plots of the residuals versus the predicted values showed no pattern. There was no multicollinearity between variables in any of the models (Supplementary Figures S1 and S2, and Table S4).

### Sensitivity analyses

Supplementary Table S5 shows the results of the univariable regressions. The robust and ordinary least squares regression models (Supplementary Tables S6 and S7) identified small negative associations between list size and male life expectancy. In the robust model, increased numbers of receptionists per 1000 patients only predicted lower female life expectancy, and the percentage of patients being seen on the same day was not a predictor.

Findings were similar for both 0% and 0.5% replacing unreported small values in White ethnicity (Supplementary Table S8). The models including different ethnicity variables were broadly similar; the ethnic groups Black, Asian, and other were positive predictors, but mixed was a positive predictor for females only (Supplementary Tables S9 and S10).

## Discussion

### Summary

Deprivation and regions were powerful predictors of life expectancy, and several practice characteristics were definite predictors but accounted for less variation. The results supported all three of the hypotheses and reflected the mechanisms of primary care set out in the conceptual framework.^13^

Greater funding was associated with higher life expectancy. More fully qualified FTE GPs predicted higher life expectancy, but numbers of GP registrars, ANPs, and receptionists did not (hypothesis 1). More nurses (excluding ANPs) predicted lower life expectancy, reflecting the employment of more practice nurses per unit of population in deprived localities.^22^ Better same-day access and continuity predicted higher life expectancy (hypothesis 2), as did some measures of clinical care (hypothesis 3), although a higher percentage of people aged ≥45 years who had a blood pressure reading recorded predicted lower life expectancy in females, possibly because people with chronic conditions were more likely to consult and, thus, have incidental blood pressure checks.^23^

### Strengths and limitations

The study was cross-sectional and limited to describing associations at the practice level. Inferences about causation based on this study should be avoided. Reliance on publicly available data restricted variable selection, and changes to some GPPS and QOF variable definitions during the quinquennium of interest limited the years that could be used.

Nonetheless, most practices existing in 2022 were included and the regression models explained a high proportion (72%–79%) of variations in life expectancy between practices. Unmeasured factors will explain some of the remaining variation. Features of secondary care or public health that affect life expectancy were not included.12,13 Both primary and secondary care have been shown to influence mortality rates of some groups of patients admitted to hospital.^24^

GP supply is influenced by a variety of factors,^22^ but in the current study deprivation, region, other population characteristics, and payments to practices were adjusted for, and the findings are consistent with those of studies of various designs from other countries.^13^ An important strength is the derivation of independent variables from evidence-based frameworks for explaining primary care’s effects on population mortality. Variables relevant to many of the mechanisms accounting for these effects were included in the current study, with each mechanism being linked via the frameworks to relevant evidence.^12^^,^^13^

The study period was pre-pandemic. Practice-level life expectancy data for a longitudinal study beginning before and continuing beyond the pandemic are not yet available, but male life expectancy in England fell during the pandemic.^1^ Future research will be needed to investigate general practice’s influence during and following the pandemic.

### Comparison with existing literature

The association between greater deprivation and higher mortality is well established, and the variation of mortality rates between regions has been reported before.^25^ Access^26^ and continuity of care^27^^,^^28^ have been shown to be associated with variations in mortality.

Studies from several countries have demonstrated links between GP supply and population mortality,^13^^,^^29^ but evidence from England is limited. Past research had suggested no association.^30^^,^^31^ However, the authors’ previous study using mortality data for 2006–2010 found an association,^8^ confirmed in this study, although the current study found no association between life expectancy and numbers of GP registrars, ANPs, or receptionists. The possible effects on mortality of these staff types are seldom investigated. The numbers of GP registrars and ANPs are relatively small, and their distributions are very skewed, making it difficult to identify any associations with mortality. Further research into this is needed. The effect of funding for general practice on life expectancy is also rarely studied,^32^ but the current study’s findings reflect international evidence that increased funding improved outcomes,^13^ and the suggestion from an economic analysis in England that investment in primary care can benefit mortality.^33^

Previous studies have shown that QOF performance has little effect on mortality,^34^^,^^35^ although a study limited to people with diabetes found an association.^36^ Two of the four QOF variables in the model in the current study were associated with variations in life expectancy, but why this differs from other studies is unclear. The finding in the current study suggests that further research is required into the potential effects of reducing or withdrawing the QOF scheme before any policy decisions.^37^

### Implications for research and practice

Deprivation and regional variations are important influences on life expectancy. General practice factors have smaller effects but, importantly, many are modifiable. The findings in the current study support increased funding and greater GP staffing of practices, but policies should also attend closely to the needs of deprived practices, and aim to improve access, continuity, and aspects of clinical care. A recent House of Commons report acknowledged that the number of FTE GPs is insufficient and declining, and that access and continuity had declined.^37^ It is thus worrying that the findings in the current study, although cross-sectional, suggest that these declines may have adversely affected life expectancy, even before the pandemic.

## Data

The dataset is loaded into Zenodo, and is publicly available via that repository (DOI: https://doi.org/10.5281/zenodo.10863649).
